# Cerebral spectroscopic and oxidative stress studies in patients with schizophrenia who have dangerously violently offended

**DOI:** 10.1186/1471-244X-8-S1-S7

**Published:** 2008-04-17

**Authors:** Ian H Treasaden, Basant K Puri

**Affiliations:** 1 Three Bridges Medium Secure Unit, West London Mental Health NHS Trust, Uxbridge Road, Southall, Middlesex UB1 3EU, UK; 2 MRI Unit, MRC Clinical Sciences Centre, Imaging Sciences Department, Imperial College London, Hammersmith Hospital, Du Cane Road, London W12 0HS, UK

## Abstract

**Background:**

The aim of this study was to bring together all the results of *in vivo *studies of ethane excretion and cerebral spectroscopy in patients with schizophrenia who have dangerously seriously violently offended in order to determine the extent to which they shed light on the degree to which the membrane phospholipid hypothesis and the actions of free radicals and other reactive species are associated with cerebral pathophysiological mechanisms in this group of patients.

**Methods:**

The patients investigated were inpatients from a medium secure unit with a DSM-IV-TR diagnosis of schizophrenia. There was no history of alcohol dependency or any other comorbid psychoactive substance misuse disorder. Expert psychiatric opinion, accepted in court, was that all these patients had violently offended directly as a result of schizophrenia prior to admission. These offences consisted of homicide, attempted murder or wounding with intent to cause grievous bodily harm. Excreted ethane was analyzed and quantified by gas chromatography and mass spectrometry (*m*/*z *= 30). 31-phosphorus magnetic resonance spectroscopy data were obtained at a magnetic field strength of 1.5 T using an image-selected *in vivo *spectroscopy sequence (TR = 10 s; 64 signal averages localized on a 70 × 70 × 70 mm^3 ^voxel).

**Results:**

Compared with age- and sex-matched controls, in the patient group the mean alveolar ethane level was higher (*p *< 0.0005), the mean cerebral beta-nucleotide triphosphate was lower (*p *< 0.04) and the mean gamma-nucleotide triphosphate was higher (*p *< 0.04). There was no significant difference between the two groups in respect of phosphomonoesters, phosphodiesters or broad resonances.

**Conclusion:**

Our results are not necessarily inconsistent with the membrane phospholipid hypothesis, given that the patients studied suffered predominantly from positive symptoms of schizophrenia. The results suggest that there is increased cerebral mitochondrial oxidative phosphorylation in patients with schizophrenia who have dangerously and seriously violently offended, with an associated increase in oxygen flux and subsequent electron 'leakage' from the electron transport chain leading to the formation of superoxide radicals and other reactive oxygen species. In turn, these reactive species might cause increased lipid peroxidation in neuroglial membranes, thereby accounting for the observation of increased ethane excretion.

## Background

In their detailed review of neurobiological correlates of violent behaviour in patients with schizophrenia, Naudts and Hodgins [1] pointed out that compared with the general population, patients with, or who will develop, schizophrenia are at increased risk for violent offending and at even higher risk of committing homicide. Within a non-dualist framework, it is clearly of more than just passing academic interest to try to establish which cerebral pathophysiological mechanisms, if any, are associated with serious and dangerous acts of violence in patients with schizophrenia. In this context, it is interesting to note that the late David Horrobin put forward his innovative membrane phospholipid hypothesis particularly with schizophrenia in mind; subsequent studies have indicated that this hypothesis may be of relevance not only to schizophrenia but also to several other disorders in psychiatry and neurology [2]. Another potentially fruitful avenue to explore is the possibility that free radicals and other reactive species are involved; they have already been implicated in over 150 human disorders, including schizophrenia [3,4].

To the best of our knowledge, our group is the only one which has investigated the possibility that these two theoretical strands may be of relevance to the group of patients with schizophrenia who have seriously and dangerously violently offended. At the level of neuroglial cell membranes, these two strands converge: increased levels of free radicals can lead to lipid peroxidation, which will remove long-chain polyunsaturated fatty acids from the Sn2 position of neuroglial cell membrane phospholipid molecules, leaving them deficient in these fatty acids as hypothesized by the membrane phospholipid hypothesis. It is fortunate that it is now possible to study both oxidative stress and the membrane hypothesis in humans by non-invasive techniques, namely expired ethane and cerebral spectroscopy. We have indeed used both these techniques in patients with schizophrenia who have dangerously and seriously violently offended.

The aim of this study was to bring together all the results of *in vivo *studies of ethane excretion and cerebral spectroscopy in patients with schizophrenia who have dangerously and seriously violently offended in order to determine the extent to which they shed light on the degree to which the membrane phospholipid hypothesis and the actions of free radicals and other reactive species are associated with cerebral pathophysiological mechanisms in this group of patients.

## Methods

### Database searches

Extensive searches of major databases, including, but not limited to, PubMed, revealed that our group was the only one to have published papers relating to the membrane phospholipid hypothesis and the actions of free radicals and other reactive species in patients with schizophrenia who have dangerously and seriously violently offended. Perusal of abstracts submitted to recent international conferences on brain lipids, schizophrenia and brain and behaviour also revealed the same.

### Subjects

The patients included by our group in those papers germane to the present study consisted of psychiatric inpatients from a medium secure unit with a diagnosis of schizophrenia according to DSM-IV-TR. Expert psychiatric opinion, accepted in court, was that all these patients had violently offended directly as a result of schizophrenia prior to admission. These offences consisted of homicide, attempted murder or wounding with intent to cause grievous bodily harm. There was no history of alcohol dependency or any other comorbid psychoactive substance misuse disorder for any of the patients.

In comparisons with normal controls, these were healthy volunteers who had no history of a psychiatric or neurological disorder, including no history of alcohol dependency or any other comorbid psychoactive substance misuse disorder, and no history of violent offending.

All investigations were carried out according to the Declaration of Helsinki, with approval from the local research ethics committee. The subjects were given details of the study and gave written informed consent.

### Exhalant analysis

Each subject was asked to exhale through a disposable sterile mouthpiece into a syringe (Markes International Ltd., UK) in one long breath, until they were no longer able to exhale any further. This enabled alveolar (end expired) air to be collected from the lungs. The air sample was then injected into an automated thermal desorption tube packed with carbotrap 300 (Perkin-Elmer, UK) via a sodium sulphate drying cartridge (International Sorbent Technology, UK). The air samples were analyzed using a Perkin-Elmer autosystem XL equipped with a turbo mass spectrometer. The automated thermal desorption tubes were desorbed onto the cold trap at 320°C, with the cold trap temperature being held at 5°C. The trap was then rapidly heated to 350°C and the liberated volatiles injected onto a 30 m × 0.32 mm PLOT GQ column (Perkin-Elmer, UK) with helium gas at 2 ml min^-1^. The oven was set at 45°C for 10 min and ramped at 14°C min^-1 ^to 200°C at which temperature it was held for 120 s. Ethane (C_2_H_6_) was eluted at 2.6 min and identified and quantified by mass spectrometry at an *m*/*z *value of 30 by comparison with a standard curve (0–60 pmol) constructed from a C1–C6 alkane standard mix (Supelco, UK).

Inter-assay variability was 17% and intra-assay variability was 10%. It was found that after one week tubes retained 97% ethane; our samples were analyzed within one week of collection.

### Cerebral spectroscopy

31-phosphorus magnetic resonance spectroscopy data were obtained using a 1.5 T Marconi Eclipse system (Marconi Medical Systems, Cleveland, Ohio). The subjects were scanned with a birdcage quadrature head coil dual-tuned to proton (^1^H, 64 MHz) and ^31^P (26 MHz). This enabled T_1_-weighted structural scans to be carried out, using the proton signal, for the purpose of spectral localization. With the subjects still in the scanner with the same head coil, the signals were switched to ^31^P, to allow ^31^P magnetic resonance spectroscopy data to be collected from the brain. Spectra were obtained using an image-selected *in vivo *spectroscopy sequence (ISIS) with a repetition time of 10 s with 64 signal averages localized on a 70 × 70 × 70 mm^3 ^voxel. Owing to the low abundance of ^31^P compared with ^1^H, the maximum size voxel was used to collect signal from the brain and thus maximize the signal-to-noise ratio.

Seven sets of peaks are characteristically identifiable in the 31-phosphorus spectrum from a normal human brain. In order of decreasing chemical shift, these peaks were assigned to phosphomonoesters, inorganic phosphate, phosphodiesters, phosphocreatine and gamma-, alpha- and beta-nucleotide triphosphate. The quantification of the ^31^P signals, using prior knowledge was carried out in the temporal domain using the AMARES algorithm [5] included in the MRUI software program [6].

### Statistical analyses

The statistical analyses of the exhalant ethane and cerebral spectroscopy data were carried out using the SPSS version 12 statistics program (SPSS Inc., Chicago).

## Results

### Exhalant analysis

The mean level of ethane in alveolar air samples from 20 patients with schizophrenia who had dangerously and seriously violently offended was higher than that of 23 age- and sex-matched healthy control subjects (*p *< 0.0005) [4].

### Cerebral spectroscopy

The mean cerebral beta-nucleotide triphosphate (*β*NTP) was lower (*p *< 0.04) and the mean gamma-nucleotide triphosphate (*γ*NTP) was higher (*p *< 0.04) in 15 male patients with schizophrenia who had dangerously and seriously violently offended than in 13 age- and sex-matched healthy control subjects [7]. There was no significant difference between the two groups in respect of phosphomonoesters or phosphodiesters. In a further study, there was no significant difference in the broad resonances in the cerebral 31-phosphorus spectra between 15 male patients with schizophrenia who had dangerously and seriously violently and 12 age- and sex-matched healthy control subjects [8].

### Ethane and cerebral spectroscopy

Ethane and percentage phosphodiester levels were positively correlated (*r*_*s *_= 0.714, *p *< 0.05) in eight patients with schizophrenia who had dangerously and seriously violently offended [9].

## Discussion

Our finding of a highly significant increase in alveolar expired ethane in patients with schizophrenia who have dangerously and seriously violently offended is consistent with increased oxidative (and/or other reactive species-related) stress in this patient group. Although, as with schizophrenia generally, cigarette smoking is relatively common in such patients, we can rule out smoking as a cause of increased alveolar ethane from a recent study by our group [10].

Turning now to the membrane phospholipid hypothesis, if this applied to the patient group studied, then we might have expected to have found changes in the phosphomonoesters, which index membrane phospholipid anabolism and/or in the phosphodiesters, which index membrane phospholipid catabolism. This was not so. And yet, we did find evidence that alveolar ethane is significantly and positively correlated with cerebral phosphodiesters. Furthermore, since the broad resonance underlying the 31-phosphorus spectrum from the living mammalian brain is composed of signal directly from cell membrane phospholipids, we would have expected to have seen changes here. Again, this was not so.

Therefore, overall our findings favour further research into the actions of free radicals and other reactive species with respect to cerebral pathophysiological mechanisms in this group of patients, but they appear to be inconsistent with the membrane phospholipid hypothesis. We should not reject the membrane phospholipid hypothesis too rapidly, however. It is noteworthy that erythrocyte membrane fatty acid changes in schizophrenia have been found relatively consistently, and tend to point to reduced levels of the long-chain polyunsaturated fatty acids arachidonic acid and docosahexaenoic acid, in the context of a biphasic distribution with one group of patients having essentially normal fatty acid levels, and the other having significantly low levels [11]. When the patients were divided into those with predominantly negative and predominantly positive syndromes, the low erythrocyte membrane levels of arachidonic acid and docosahexaenoic acid were largely confined to patients with a negative syndrome [2]. All the patients with schizophrenia who have dangerously and seriously violently offended and who took part in the present set of studies fit into the category of a positive syndrome. As we have commented elsewhere [8], this may well explain why our results were at variance with the cerebral spectroscopy changes that we might have expected from the membrane phospholipid hypothesis.

Our cerebral spectroscopy findings of lower *β*NTP and higher *γ*NTP in the patient group, compared with the healthy controls, are of interest. It is well established that adenosine triphosphate (ATP) is the most widely used activated carrier molecule; under normal circumstances, when the brain requires energy, this can readily be obtained by the energetically favourable hydrolysis of ATP to adenosine diphosphate (ADP) and inorganic phosphate. While *β*NTP indexes ATP, the majority of ADP is nuclear magnetic resonance (and therefore neurospectroscopy)-invisible; the *γ*NTP signal does overlap with signals from *β*ADP [12], although the signal of *γ*NTP is much stronger. Therefore, our results are consistent with increased energy metabolism taking place in this patient group. This suggests that there may be a change in mitochondrial function in the brain in such patients.

Overall, our results are consistent with there being increased mitochondrial oxidative phosphorylation in the brain in the patient group studied, with an associated increase in oxygen flux and subsequent electron 'leakage' from the electron transport chain, thereby forming superoxide radicals and other reactive oxygen species, as has been demonstrated in the mammalian heart during exercise [13,14].

## Conclusion

Our results suggest that there is increased cerebral mitochondrial oxidative phosphorylation in patients with schizophrenia who have dangerously and seriously violently offended, with an associated increase in oxygen flux and subsequent electron 'leakage' from the electron transport chain leading to the formation of superoxide radicals and other reactive oxygen species. In turn, these reactive species might cause increased lipid peroxidation in neuroglial membranes, thereby accounting for the observation of increased ethane excretion.

## Competing interests

The author(s) declare that they have no competing interests.

## Authors' contributions

IHT and BKP made substantial contributions to the conception and design of this work, and to the analysis and interpretation of the data. Both authors have also been involved in drafting and revising the manuscript and have read and approved the final manuscript.
